# A Study on How Methionine Restriction Decreases the Body’s Hepatic and Lipid Deposition in Rice Field Eel (*Monopterus albus*)

**DOI:** 10.3390/ijms222413379

**Published:** 2021-12-13

**Authors:** Yajun Hu, Minglang Cai, Huan Zhong, Wuying Chu, Yi Hu

**Affiliations:** 1 Hunan Engineering Technology Research Center of Featured Aquatic Resources Utilization, Hunan Agricultural University, Changsha 410128, China; Huyajun@stu.hunau.edu.cn (Y.H.); mineraltsai0518@163.com (M.C.); zhonghuan@hunau.edu.cn (H.Z.); 2 College of Animal Science and Technology, Hunan Agricultural University, Changsha 410128, China; 3 Department of Bioengineering and Environmental Science, Changsha University, Changsha 410000, China; chuwuying18@163.com

**Keywords:** methionine, hepatic structure, hepatic lipid metabolism, *Monopterus albus*

## Abstract

Methionine restriction reduces animal lipid deposition. However, the molecular mechanism underlying how the body reacts to the condition and regulates lipid metabolism remains unknown. In this study, a feeding trial was performed on rice field eel *Monopterus albus* with six isonitrogenous and isoenergetic feeds that included different levels of methionine (0, 2, 4, 6, 8, and 10 g/kg). Compared with M0 (0 g/kg), the crude lipid and crude protein of *M. albus* increased markedly in M8 (8 g/kg) (*p* < 0.05), serum (total cholesterol, triglyceride, high-density lipoprotein cholesterol, low-density lipoprotein cholesterol, and non-esterified free fatty acids), and hepatic contents (hepatic lipase, apolipoprotein-A, fatty acid synthetase, total cholesterol, triglyceride, and lipoprteinlipase). However, in the serum, very-low-density lipoprotein and hepatic contents (hormone-sensitive triglyceride lipase, Acetyl CoA carboxylase, carnitine palmitoyltransterase, and mirosomal triglygeride transfer protein) decreased markedly in M8 (*p* < 0.05). The contents of hepatic C18:2n-6, C22:6n-3, and n-3PUFA in the M8 group were significantly higher than those in M0 (*p* < 0.05), and the contents of lipid droplets in M8 were higher than those in M0. Compared with M0, the hepatic gcn2, eif2α*, hsl, mttp, ldlrap, pparα, cpt1*, and *cpt2* were remarkably downregulated in M8, while *srebf2, lpl, moat2, dgat2, hdlbp, srebf1, fas, fads2, me1, pfae,* and *icdh* were markedly upregulated in M8. Moreover, hepatic SREBP1 and FAS protein expression were upregulated significantly in M8 (*p* < 0.01). In short, methionine restriction decreased the lipid deposition of *M. albus*, especially for hepatic lipid deposition, and mainly downregulated hepatic fatty acid metabolism. Besides, *gcn2* could be activated under methionine restriction.

## 1. Introduction

Recently, studies reported that soybean protein can be used to replace fish meal (FM) in aquatic feed [1]. However, methionine is the most limiting amino acid in soybean protein, and essential sulfur amino acids for fish [2] must be obtained from feed [3]. Methionine not only participate in the body’s protein synthesis but also directly or indirectly (through transsulfuration, transamination, and transmethylation) regulates the body’s metabolism as a signal molecule, mainly metabolizing into cysteine, creatine, carnitine, hydrogen sulfide, taurine, and glutathione for various metabolic purposes [4]. If an aquatic animal’s methionine intake is deficient, the process of protein synthesis will be limited, metabolism will be disturbed, and the growth performance of the fish will be inhibited [2,5,6].

A previous study showed that methionine restriction enhances the clearance of glucose, promotes hepatic fat accumulation, and decreases muscular fat accumulation in rainbow trout (Oncorhynchus mykiss) [7]. In addition, methionine restriction suppresses the targets of amino acid response pathways in the primary muscular cells of turbot *(Scophthalmus maximus* L.), reduces cellular protein synthesis, enhances protein degradation, increases levels of intracellular free amino acid, and leads to amino acid degradation. Methionine restriction also reduces glycolysis and lipogenesis while stimulating lipolysis, decreases the intracellular lipid pool, remarkably enhances energy expenditure by stimulating the tricarboxylic acid cycle and oxidative phosphorylation, and upregulates general controlled nonderepressible 2 (gcn2, also encoded by eif2ak4) expression [8].

In the process of evolution, animals have gradually evolved the ability to adapt to a lack of essential nutrients such as essential amino acids. In vertebrates, gcn2 plays a key role in sensing essential amino acid deprivation and activates the translational derepression of specific mRNAs by inhibiting general translation initiation [9]. A study on the Cobia (Rachycentron canadum) showed that crude lipids were markedly elevated with a higher level of dietary methionine and then plateaued. Hepatic lipid synthesis genes (sterol regulatory element binding protein-1 (srebp1), fatty acid synthetase (fas), peroxisome proliferator activated receptor γ (pparγ), and stearoyl-CoA desaturase-1 (scd1)) were significantly upregulated when the animals were fed a diet with higher levels of methionine, whereas the expression of lipolytic genes (peroxisome proliferator activated receptor α (pparα), carnitine acyl transferase-1 (cpt1), and lipase lipoprotein lipase (lpl)) was elevated in fish fed a methionine-deficient diet [10]. Guo et al. (2007) found that the adipose tissue of wild-type mice lacking leucine decreased by 50% after one week and almost completely disappeared after 17 days. Further research found that when leucine was deficient in the diet, gcn2 was activated, and its downstream eif2α, the level of mRNA, and protein expression increased. However, there was no significant difference observed in the expression of srebp1a and srebp2 mRNA and protein, although srebp1c mRNA and protein expression were significantly inhibited. Moreover, the expression of srebp1c mRNA and protein was regulated by the gcn2-eif2α pathway. The expression of fat synthesis genes (srebp1c, ATP-citrate lyase (acl), fas, scd, glucose 6-phosphate 1-dehydrogenase (g6pd), and malic enzyme (me)) occurred downstream, and hepatic SREBP1 and FAS protein expression was downregulated. The authors also found that a diet lacking leucine led to an increase in lipid absorption and fatty acid oxidation in the livers of mice, suggesting that the increase of lipid absorption and decomposition in mice under the condition of leucine deficiency was an adaptive change to reduce lipid synthesis [11].

Rice field eel (*Monopterus albus*, M. albus) is a subtropical freshwater benthic fish that is widely raised in central and southern China in cages [12]. Our previous studies showed that M. albus needs better-quality and higher levels of protein, as well as an optimum protein/lipid ratio [13]. In the study, FM was replaced by soybean meal [14], and soy protein concentrate inhibited the growth performance of M. albus [15]. Moreover, dietary deficiency methionine feed decreased the growth performance of M. albus, induced lipid metabolism disorder, and decreased lipid content [16]. Our laboratory is focused on studying the nutrition of M. albus. We also consulted a large number of papers of M. albus and found no obvious adipose tissue in M. albus. Lipids mainly accumulated in tissues, especially in the liver, which provides a new and interesting avenue for exploring lipid metabolism. In the present study, we generated more severe methionine-deficient diets compared to our previous study [16] and explored the mechanism by which methionine regulates lipid deposition and the metabolism of M. albus.

## 2. Results

### 2.1. Composition of M. Albus

There was no significant difference in the moisture and crude ash of *M. Albus* among all groups (*p* > 0.05). The crude lipid of *M. albus* increased markedly as methionine concentrations increased to 8 g/kg (M8) (*p* < 0.05) and gradually decreased as methionine concentrations increased to 10 g/kg (M10). The crude protein of *M. albus* increased markedly as methionine concentrations were increased (*p* < 0.05) (Table 1).

### 2.2. Serum Biochemical Indices

Serum ACP, Glu, TC, TG, HDL, LDL, and NEFA increased markedly as methionine concentrations increased to 8 g/kg (M8) (*p* < 0.05) and gradually decreased as methionine concentrations increased to 10 g/kg (M10). The serum TP, BUN, and Ba also increased markedly as methionine concentrations increased to 10 g/kg (M10) (*p* < 0.05). Serum AKP, ALT, AST, and VLDL decreased markedly as methionine concentrations increased to 8 g/kg (M8) (*p* < 0.05) and gradually increased as methionine concentrations increased to 10 g/kg (M10) (Table 2).

### 2.3. Hepatic Biochemical Indices

Compared to M0 (0 g/kg), the hepatic HL, Apo-A, FAS, TC, and AKP increased markedly under methionine supplementation (*p* < 0.05). Moreover, hepatic TG and ALT increased markedly under greater than 2 g/kg methionine supplementation (*p* < 0.05), the hepatic AST increased markedly with more than 4 g/kg methionine supplementation (*p* < 0.05), and hepatic LPL increased markedly in the M6 (6 g/kg) and M8 (8 g/kg) groups (*p* < 0.05). Compared to M0 (0 g/kg), the hepatic HSL and ACC decreased markedly when methionine was added (*p* < 0.05); the hepatic CPT decreased markedly with greater than 2 g/kg methionine (*p* < 0.05); and the hepatic MTTP decreased markedly in M2 (2 g/kg), M6 (6 g/kg), M8 (8 g/kg), and M10 (10 g/kg) (*p* < 0.05) (Table 3).

### 2.4. Contents of Hepatic Amino Acids and Fatty Acids

Compared to M0 (0 g/kg), the contents of hepatic amino acids, total essential amino acids, total nonessential amino acids, and total amino acids increased gradually under supplementation with 8 g/kg (M8) methionine (Table 4). Moreover, compared to M0 (0 g/kg), the hepatic C18:2n-6, C22:6n-3, and n-3PUFA significantly increased in the M8 (8 g/kg) group (*p* < 0.05) (Table 5).

### 2.5. Hepatic H&E and Oil Red O-Stained Pictures

**Figure 1:**
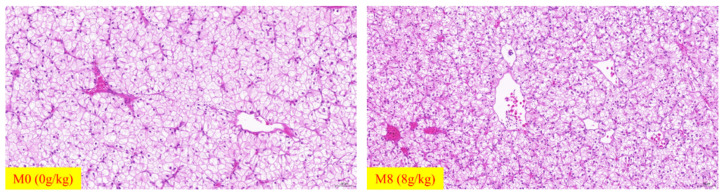
Effects of deficient and optimum methionine diets on hepatic H&E-stained images (×200) of *M. Albus* after 8 weeks.

**Figure 2:**
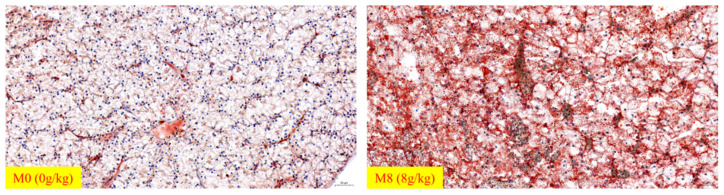
Effects of deficient and optimum methionine diets on hepatic oil red-O-stained images (×200) of *M. Albus* after 8 weeks.

Hepatic H&E and Oil red O-stained images are shown in Figure 1 and Figure 2. Vacuoles were observed in these two groups. As the number of vacuoles increased in the M0 group, the proportions of vacuoles decreased in the M8 (8 g/kg) group. We also observed movement of the nucleus in M0 (0 g/kg) and blurred boundaries of hepatic cells in the M0 group. Compared to M0 (0 g/kg), the number of lipid droplets was increased in the M8 (8 g/kg) group (Figure 1 and Figure 2).

### 2.6. Hepatic Lipid Metabolism mRNA Expression

**Figure 3:**
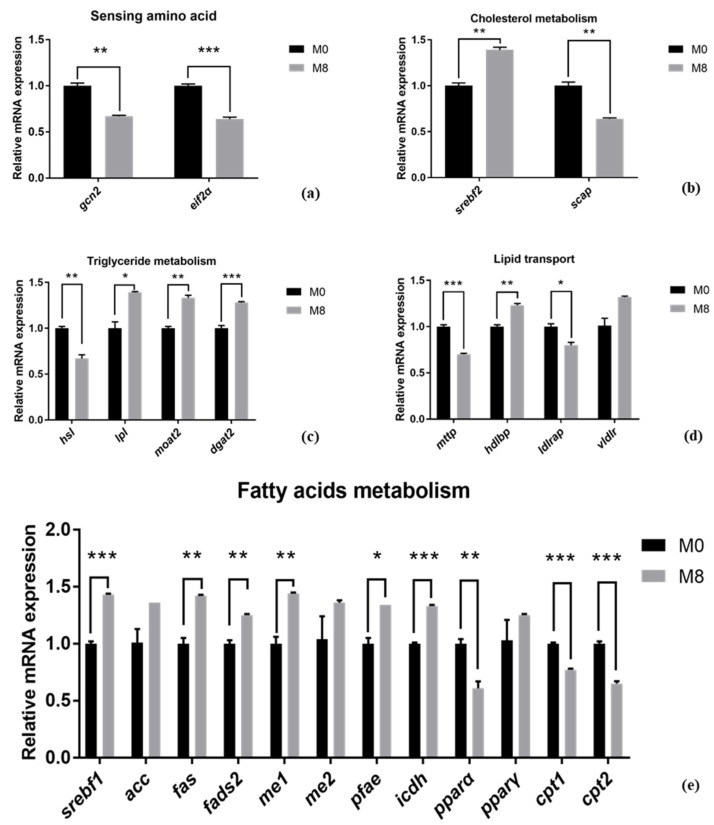
Effects of dietary methionine on the hepatic lipid metabolism mRNA expression of *M. Albus* after 8 weeks (*n* = 3). Single, double, or triple numbers of asterisks were significantly different at *p* < 0.05, *p* < 0.01 and *p* < 0.001, respectively.

Compared to M0 (0 g/kg), the hepatic *gcn2*, *eif2α*, *hsl*, *mttp*, *ldlrap*, *pparα*, *cpt1,* and *cpt2* were remarkably downregulated in M8 (8 g/kg) (*p* < 0.01, *p* < 0.001, *p* < 0.01, *p* < 0.001, *p* < 0.05, *p* < 0.01, *p* < 0.001, and *p* < 0.001, respectively). However, *srebf2*, *lpl*, *moat2*, *dgat2*, *hdlbp*, *srebf1*, *fas*, *fads2*, *me1*, *pfae,* and *icdh* were markedly upregulated in M8 (8 g/kg) (*p* < 0.05, *p* < 0.01, *p* < 0.001, *p* < 0.05, *p* < 0.01, *p* < 0.001, *p* < 0.01, *p* < 0.001, *p* < 0.01, *p* < 0.01, *p* < 0.01, *p* < 0.05, and *p* < 0.001, respectively) (Figure 3).

### 2.7. Correlative Analysis of Hepatic Lipid Metabolism Gene Expression

**Figure 4:**
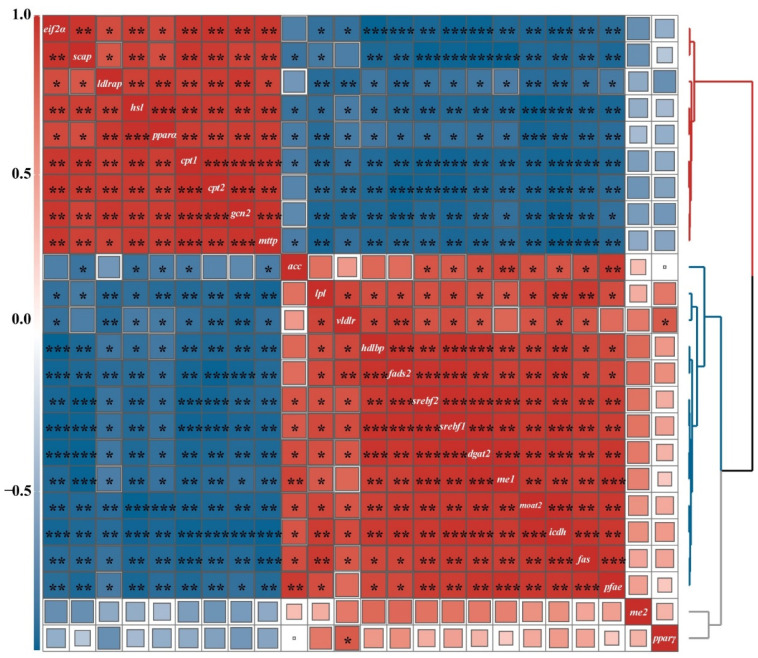
Correlative analysis of hepatic lipid metabolism gene expression performed using the R Programming Language. Single, double, and triple asterisks were significantly different at *p* < 0.05, *p* < 0.01, and *p* < 0.001, respectively.

We observed that hepatic *eif2α*, *scap*, *hsl*, *mttp*, *ldlrap*, *pparα*, *cpt1,* and *cpt2* gene expression was positively correlated with *gcn2* (*p* < 0.01, *p* < 0.01, *p* < 0.01, *p* < 0.001, *p* < 0.01, *p* < 0.01, *p* < 0.001, and *p* < 0.001, respectively), while hepatic *srebf2*, *lpl*, *moat2*, *dgat2*, *hdlbp*, *vldlr*, *srebf1*, *fas*, *fads2*, *me1*, *pfae*, and *icdh* gene expression was negatively correlated with *gcn2* (*p* < 0.01, *p* < 0.01, *p* < 0.01, *p* < 0.01, *p* < 0.01, *p* < 0.01, *p* < 0.01, *p* < 0.001, *p* < 0.001, *p* < 0.05, *p* < 0.05, and *p* < 0.001) (Figure 4).

### 2.8. Hepatic SREBP1 and FAS Protein Expression

**Figure 5:**
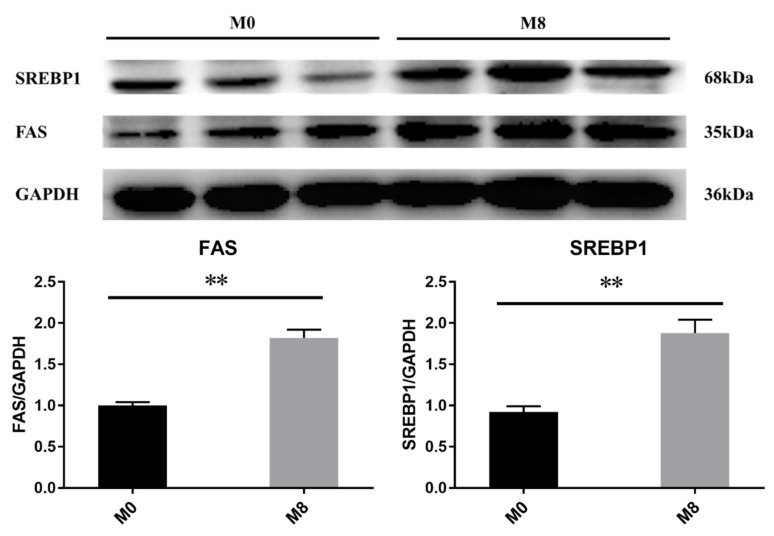
Effects of dietary methionine on the hepatic lipid metabolism protein expression of *M. Albus* after 8 weeks (*n* = 3). Double asterisks were significantly different at *p* < 0.01, respectively.

Compared to M0, both hepatic SREBP1 and FAS protein expression was upregulated significantly in M8 (8 g/kg) (*p* < 0.01) (Figure 5).

## 3. Discussions

Our previous study showed that dietary methionine restriction induced lipid metabolism disorder, decreased the lipid content [16], and also decreased the growth performance of *M. Albus* [17]. In the present study, the crude lipids of *M. albus* increased markedly as methionine concentrations increased to 8 g/kg and gradually decreased as methionine concentrations increased to 10 g/kg. The crude protein of *M. albus* increased markedly as methionine concentrations were increased. Our results are similar to those of a study on juvenile yellow tail (*Seriola dorsalis*) [18]. We inferred that more energy was allocated to visceral organs to maintain basic metabolism while fewer nutrients were allocated to growth performance when methionine was restricted.

Amino acids are commonly involved in life activities through the synthesis of proteins. Excess amino acids are generally decomposed into ammonia and carbon skeletons, while ammonia is further metabolized into urea nitrogen [19]. The acid phosphatase (ACP) enzyme is involved in protein pinocytosis and intracellular digestion [20]. Alkaline phosphatase (AKP) is a key enzyme with a protective role in fish under stress, parasitic infection, and wound healing [21]. In this study, serum ACP increased markedly when supplemented with a suitable level of methionine (8 g/kg), the serum AKP decreased markedly as methionine concentrations increased to 8 g/kg, and hepatic AKP significantly increased when supplemented with methionine. Transaminases are produced by the liver. Aspartate aminotransferase (AST) primarily transfers the amino of aspartic acid to a-ketone glutaric acid, producing oxaloacetic acid and glutamic acid, while alanine aminotransferase (ALT) primarily transfers the amino of alanine to a keto-glutamic acid, producing pyruvate and glutamic acid; these acids are also the main indexes used to evaluate hepatic injury [22]. In this study, the serum ALT and AST decreased markedly under supplementation with methionine (8 g/kg). Meanwhile, the hepatic ALT and AST increased markedly when supplemented with methionine concentrations greater than 4 g/kg. Moreover, the serum total protein, blood urea nitrogen, and blood ammonia increased markedly when supplemented with a suitable level of methionine (8 g/kg). The contents of hepatic amino acids, total essential amino acids, total nonessential amino acids, and total amino acids also increased. This phenomenon increased the utilization efficiency of amino acid. In this study, we also observed that the proportions of vacuoles decreased under supplementation with methionine (8 g/kg). Meanwhile, the nucleus moved and blurred the boundaries of hepatic cells when methionine was restricted. We concluded that suitable methionine may be better for hepatic amino-acid metabolism and a healthy condition, as we reported in [16].

Interestingly, we also observed that the serum glucose, total cholesterol, and triglycerides increased significantly with 8 g/kg dietary methionine. Meanwhile, the hepatic total cholesterol and triglycerides increased markedly when supplemented with higher than 2 g/kg methionine in this study. High-density lipoprotein (HDL) and low-density lipoprotein (LDL) are major lipoproteins produced by the liver. LDL transports lipid molecules from the liver around the body, while HDL carries lipids from the surrounding tissue into the liver. These lipoproteins mainly carry cholesterol and are formed as HDL-C and LDL-C, respectively [23]. Very-low-density lipoprotein (VLDL) is secreted by hepatocytes of the liver; the large sizes of VLDL particles secreted by the liver result in major disturbances to lipoprotein metabolism [24]. Hormone-sensitive lipase (HSL) regulates lipolysis, especially in adipose tissue [25]. Microsomal triglyceride transfer protein (MTP) facilitates the transport of fat by assisting in the assembly and secretion of triglyceride-rich apolipoprotein [26]. Apolipoprotein A-1 (ApoA1) is considered to be an important factor in lipid transport and metabolism in various tissues [27]. Lipoprotein lipase (LPL) is a key enzyme in lipid metabolism and primarily catalyzes the hydrolysis of triglycerides in chyle particles and very-low-density lipoprotein [28]. Fatty acid synthase (FAS) is involved in fatty acid synthase [29], and Acetyl-CoA carboxylase (ACC) is the rate-limiting enzyme for fatty-acid synthesis [30]. Carnitine palmitoyltransterase (CPT) participates in the process of fatty acid β-oxidation [31]. In the present study, hepatic HL, Apo-A, FAS, and LPL increased markedly when supplemented with methionine, while hepatic HSL, ACC, CPT, and MTTP decreased markedly when methionine was added. This phenomenon indicated that methionine restriction not only inhibited amino-acid metabolism but also disturbed lipid metabolism. Our results showed that dietary methionine offers benefits for lipid metabolism. This phenomenon is similar to that observed in Cobia (*Rachycentron canadum*) [10].

In addition, the lipid droplets (visualized by hepatic Oil red O staining) was increased in the group supplemented with methionine (8 g/kg). This result intuitively shows the difference in the hepatic lipid deposition of *M. Albus* between the M0 (0 g/kg) and M8 (8 g/kg) groups. To further explain the reasons why methionine deficiency affects the lipid metabolism of *M. albus*, we chose the M0 (0 g/kg) and M8 (8 g/kg) groups to explore the molecular mechanisms of lipid metabolism. *gcn2* and *eif2a* respond to essential amino acid deprivation and regulate the downstream genes related to lipid metabolism [32]. In this study, compared to M0 (0 g/kg), hepatic *gcn2* and *eif2α* were remarkably downregulated in M8 (8 g/kg), which means that the *gcn2* and *eif2α* genes may be regulated by different levels of methionine. Thus, we determined the genes related to lipid metabolism and explored the relationship between amino-acid sensing and lipid metabolism. Sterol regulatory element binding transcription factor (*srebf*), including *srebf1* (mainly regulates fatty acids biosynthesis) and *srebf2* (mainly regulates cholesterol synthesis), controls cellular lipid metabolism and homeostasis and performs functions in lipid biosynthesis and uptake-gene expression [33]. *scap* (*srebf* cleavage-activating protein) is a sterol-regulated escort protein that transports *srebf* from its site of synthesis in the endoplasmic reticulum to its site of cleavage in the Golgi [34]. Peroxisome proliferator-activated receptor α (*pparα*) mainly controls the β-oxidation of fatty acids [35], while peroxisome proliferator-activated receptor γ (*pparγ*) regulates the adipogenic and lipogenic pathways [36]. *mogat2*, *dgat2*, *me1*, *me2*, *fas*, *fads2*, and *acc* are key enzymes involved in lipogenesis and fatty-acid synthesis [37,38,39,40,41,42,43], while *lpl*, *hsl*, *cpt1,* and *cpt2* are key genes involved in lipolysis and fatty-acid β-oxidation [44,45]. *icdh* is one of the key enzymes involved in the production of NADPH, which is an essential cofactor for fat cholesterol biosynthesis and fat metabolism [46]. The polyunsaturated fatty acid elongase (*pfae*) gene encodes desaturase and elongase enzymes with all the activities required for the production of long-chain polyunsaturated fatty acid [47]. Here, compared to M0 (0 g/kg), hepatic *pparα*, *cpt1,* and *cpt2* were remarkably downregulated in M8 (8 g/kg), while hepatic *srebf1*, *srebf2*, *lpl*, *moat2*, *dgat2*, *fas*, *fads2*, *me1*, *pfae,* and *icdh* were upregulated in M8 (8 g/kg). We also observed that lipid synthesis genes were upregulated under a dietary-suitable level of methionine, while genes related to lipid catabolism were downregulated. These phenomena observed in the present study are similar to those observed in a previous study on Cobia (*Rachycentron canadum*) [10,48] and large Yellow croaker (*Larimichthys crocea*) [49]. We also found that hepatic SREBP1 and FAS protein expression was upregulated significantly in M8 (8 g/kg). Interestingly, hepatic C18:2n-6, C22:6n-3, and n-3PUFA remarkably increased when supplemented with methionine (8 g/kg). Thus, we determined that *M. albus* dietary intake deficient in methionine mainly affected fatty-acid metabolism, specifically unsaturated fatty-acid synthesis.

Microsomal triglyceride transfer protein (*mttp*) facilitates the transport of fat by assisting in the assembly and secretion of triglyceride-rich lipoproteins [26]. High-density lipoprotein-binding protein (*hdlbp*) mainly participates in the endocrine regulation of both lipids and cholesterol [50], while low-density lipoprotein receptor adapter protein (*ldlra*) maintains levels of homeostatic LDL. Moreover, the *ldlra* pathway has emerged as a target to reduce circulating cholesterol [51]. The very-low-density lipoprotein receptor (*vldlr*) receptor binds triglyceride-rich lipoproteins, along with *lpl* [52]. In this study, hepatic *mttp* and *ldlrap* were remarkably downregulated when supplemented with methionine (8 g/kg), while *hdlbp* was up strongly regulated in M8 (8 g/kg). This result indicates that lipid metabolism is more active if the feed intake of *M. albus* features a suitable level of methionine. Moreover, hepatic *eif2α*, *scap*, *hsl*, *mttp*, *ldlrap*, *pparα*, *cpt1,* and *cpt2* gene expression was positively correlated with *gcn2*, and hepatic *srebf2*, *lpl*, *moat2*, *dgat2*, *hdlbp*, *vldlr*, *srebf1*, *fas*, *fads2*, *me1*, *pfae*, and *icdh* gene expression was negatively correlated with *gcn2*. These results indicate that *gcn2* could respond to the condition of methionine restriction in *M. albus* and regulate lipid metabolism genes. However, the specific mechanism by which *gcn2* regulates hepatic lipid metabolism requires further study.

## 4. Materials and Methods

### 4.1. Ingredients and Experimental Diets

The basic diet (110 g/kg fish meal and 400 g/kg soy protein concentrate) was based on our previous data [15,16]. Different levels of methionine (0, 2, 4, 6, 8, or 10 g/kg) were supplemented in the basic diet based on the rule of equal nitrogen and our previous studies [14,16], showed in Table 6, Table 7 and Table 8.

Proximate analysis (moisture, crude protein, crude lipid, ash, and gross energy) of experimental feed and *M. albus* was performed based on our previous papers [53]. Amino acids were analyzed by an automatic amino acid analyzer (Agilent-1100, Agilent Technologies Co., Ltd., Santa Clara, CA, USA) based on Wijerath’s method [54], and fatty acids were analyzed by GC-MS (Agilent 7890B-5977A, Agilent Technologies Co., Ltd., Santa Clara, CA, USA) based on Jin’s method [55], the results are shown in Table 2 and Table 3.

### 4.2. Fish Rearing and Management

*M. albus* was obtained from Changde, China. *M. albus* of uniform size (25.08 ± 0.31 g) was stochastically divided into 18 float cages (2.0 m × 1.5 m × 1.5 m). Each group contained triplicates with 60 fish, based on our previous study [23].

### 4.3. Ethics Statement

Our study was supported by the Animal Care Committee of Hunan Agricultural University (Changsha, Hunan, China) and conducted according to the Chinese guidelines for animal welfare. According to the guidelines established by the National Institutes of Health, all experimental fish were anesthetized with eugenol (1:12,000; Shanghai Reagent Corporation, Shanghai, China). Ethic code number: 2021094; date of Ethics Statement: 13 December 2021.

### 4.4. Sample Collection and Analyses

After the feeding trial, the caudal vein blood was heparinized from five fish in each cage. Serum (3500× *g*) was obtained by centrifugation for 10 min and then stored at −80 °C until use. Serum alanine aminotransferase, aspartate aminotransferase, acid phosphatase, alkaline phosphatase, glucose, lactate dehydrogenase, total cholesterol, triglyceride, total protein, high-density lipoprotein cholesterol, low-density lipoprotein cholesterol, non-esterified free fatty acids, blood urea nitrogen, and blood ammonia were determined by a kit from NanJing JianCheng Bioengineering (Nanjing, China). Serum very-low-density lipoproteins were determined using a kit from Shanghai Enzyme-linked Biotechnology Co., Ltd. (Shanghai, China).

Hepatic lipase, lactate dehydrogenase, microsomal triglyceride transfer protein, apolipoprotein-A, hormone-sensitive triglyceride lipase, fatty acid synthetase, lipoprotein lipase, acetyl CoA carboxylase, and carnitine palmitoyltransterase were determined using a kit from Shanghai Enzyme-linked Biotechnology Co., Ltd. (Shanghai, China). Hepatic triglyceride, total cholesterol, aspartate aminotransferase, alanine aminotransferase, alkaline phosphatase, and acid phosphatase were determined using a kit from NanJing JianCheng Bioengineering (Nanjing, China).

Hepatic amino acids were analyzed by an automatic amino acid analyzer (Agilent-1100, Agilent Technologies Co., Ltd., Santa Clara, CA, USA), and hepatic fatty acids were analyzed by GC-MS (Agilent 7890B-5977A, Agilent Technologies Co., Ltd., Santa Clara, CA, USA) using the same method.

The liver was taken from five fish per cage for histometric evaluation. The methods for creating slides and observing the muscular sections stained with H&E were based on those used in our previous paper [17]. The liver was sectioned (8 μm) using a cryostat microtome and stained with Oil Red O [56]. The slides were then observed using CaseViewer.

Total hepatic RNA was obtained from five fish per cage using the Monzol™ reagent (Monad, Shanghai, China). Smart cDNA was synthesized using a SMART cDNA Synthesis kit (Clontech Laboratories, Palo Alto, CA, USA). Primers were synthesized by Biosune Biotechnology, Inc. (Shanghai, China), as shown in Table 4. Quantitative real-time PCR (qPCR) was performed as described in our previous paper [57]. The amplification efficiency was between 0.95 and 1.10, as calculated by the formula E = 10*(−1/slope)−1, and 5-fold serial dilutions of cDNA (triplicate) were used to generate the standard curve. The 2^−^^△△Ct^ method was used to calculate the relative mRNA expression [58].

Hepatic proteins were extracted from the liver with a lysis solution. After centrifugation for 5 min at 12,000 rpm/min and 4 °C, we determined the content of protein, ensured the protein concentrations were consistent, and used the concentrations for Western blot analysis. The first antibody was as follows: GAPDH Mouse Monoclonal antibody (proteintech, catalog number: 60004-1-Ig), SREBP1 anti-Rabbit pAb (Wanleibio, WL02093), and FAS anti-Rabbit pAb (Wanleibio, WL03376). We used the ImageJ software to calculate the expression of the protein (Table 9).

### 4.5. Statistical Analysis

Data were analyzed by one-way analysis of variance (ANOVA), and significant differences among all groups were assessed by Duncan’s multiple-range test. The data of two groups (M0 & M8) were calculated by an independent T-test. The ANOVA and independent *t*-test were performed using the SPSS 22 software. The results were expressed as the means ± SEM (standard error of the mean), and differences were considered significant at *p* < 0.05.

## 5. Conclusions

Methionine restriction inhibited the lipid deposition of *M. albus*, especially for hepatic lipid deposition, and primarily downregulated hepatic fatty acid metabolism. In addition, *gcn2* was activated when methionine was restricted, and hepatic lipid-metabolism genes were correlated with *gcn2*.

## Figures and Tables

**Table 1:** Effects of different levels of methionine on the composition of *M. Albus* after 8 weeks (wet weight %).

Proximate Composition	M0	M2	M4	M6	M8	M10	*p* Value
Moisture	77.22 ± 0.44	77.48 ± 0.58	77.14 ± 0.47	77.55 ± 0.68	77.81 ± 0.69	77.2 ± 0.07	0.939
Crude ash	2.7 ± 0.01	2.73 ± 0.08	2.72 ± 0.01	2.69 ± 0.03	2.73 ± 0.04	2.71 ± 0.02	0.959
Crude lipid	1.79 ± 0.02 ^a^	2.4 ± 0.11 ^b^	2.55 ± 0.12 ^b^	2.56 ± 0.06 ^b^	3.1 ± 0.03 ^c^	2.91 ± 0.06 ^c^	<0.001
Crude protein	14 ± 0.27 ^a^	14.69 ± 0.24 ^b^	15.04 ± 0.15 ^b^	15.38 ± 0.17 ^b^	16.54 ± 0.37 ^c^	16.85 ± 0.13 ^c^	<0.001

Values are presented as the means ± SEM (*n* = 3). Values in the same row with the same superscript or the absence of superscripts are not significantly different (*p* > 0.05) (the same below).

**Table 2:** Effects of different levels of methionine on the serum biochemical indices of *M. Albus* after 8 weeks.

Index	M0	M2	M4	M6	M8	M10	*p* Value
^1^ ACP	17.95 ± 0.2 ^a^	18.46 ± 0.03 ^b^	18.61 ± 0.06 ^b^	19.23 ± 0.11 ^c^	19.42 ± 0.21 ^c^	19.42 ± 0.19 ^c^	<0.001
^2^ AKP	149.07 ± 23.6 ^b^	116.11 ± 12.57 ^a,b^	100.11 ± 2.73 ^a^	98.21 ± 2.57 ^a^	78.32 ± 3.9 ^a^	98.69 ± 17.47 ^a^	0.018
^3^ ALT	7.42 ± 0.09 ^e^	6.15 ± 0.13 ^d^	5.55 ± 0.19 ^c^	5.3 ± 0.08 ^b,c^	4.74 ± 0.06 ^a^	5.1 ± 0.05 ^b^	<0.001
^4^ AST	19.25 ± 0.34 ^d^	17.9 ± 0.07 ^c^	17.64 ± 0.17 ^c^	16.47 ± 0.27 ^b^	13.17 ± 0.09 ^a^	13.38 ± 0.4 ^a^	<0.001
^5^ Glu	2.13 ± 0.02 ^a^	2.67 ± 0.02 ^b^	2.89 ± 0.04 ^c^	3.26 ± 0.03 ^d^	3.58 ± 0.03 ^e^	3.26 ± 0.02 ^d^	<0.001
^6^ TC	3.9 ± 0.04 ^a^	3.92 ± 0.04 ^a^	4.5 ± 0.02 ^b^	4.54 ± 0.04 ^b^	4.9 ± 0.03 ^c^	4.83 ± 0 ^c^	<0.001
^7^ TG	1.04 ± 0 ^a^	1.14 ± 0.01 ^b^	1.14 ± 0.01 ^b^	1.25 ± 0.01 ^c^	1.62 ± 0.02 ^d^	1.27 ± 0.01 ^c^	<0.001
^8^ TP	43.05 ± 0.27 ^a^	46.01 ± 0.31 ^b^	46.82 ± 0.09 ^b,c^	47.4 ± 0.54 ^c^	50.62 ± 0.31 ^d^	51.03 ± 0.42 ^d^	<0.001
^9^ HDL	1.55 ± 0.04 ^a^	1.75 ± 0.06 ^a^	2.5 ± 0.32 ^b^	2.76 ± 0.3 ^b^	2.94 ± 0.24 ^b^	2.78 ± 0.27 ^b^	<0.001
^10^ LDL	0.4 ± 0.03 ^a^	0.42 ± 0.03 ^a^	0.45 ± 0.05 ^a^	0.83 ± 0.05 ^b^	1.04 ± 0.05 ^c^	1.02 ± 0.05 ^c^	<0.001
^11^ VLDL	3.06 ± 0.13 ^d^	2.69 ± 0.1 ^c^	2.44 ± 0.14 ^c^	1.93 ± 0.08 ^b^	1.29 ± 0.1 ^a^	1.82 ± 0.04 ^b^	<0.001
^12^ NEFA	87.93 ± 3.78 ^a^	90.5 ± 2.52 ^a^	94.94 ± 6.42 ^a^	118.69 ± 5.43 ^b^	136.29 ± 4.23 ^b^	121.34 ± 9.8 ^b^	<0.001
^13^ BUN	1.09 ± 0.02 ^a^	1.16 ± 0.05 ^ab^	1.35 ± 0.05 ^b,c^	1.43 ± 0.07 ^c^	1.47 ± 0.08 ^c^	1.49 ± 0.12 ^c^	0.001
^14^ Ba	166.08 ± 1.5 ^a^	183.37 ± 2.52 ^ab^	197.91 ± 2.1 ^b,c^	199.19 ± 2.7 ^b,c^	212.55 ± 7.35 ^c^	215.05 ± 12.36 ^c^	<0.001

^1^ ACP: Acid phosphatase (g/L). ^2^ AKP: Alkaline phosphatase (mg/L). ^3^ ALT: Alanine aminotransferase (u/L). ^4^ AST: Aspartate aminotransferase (u/L). ^5^ Glu: Glucose (mmol/L). ^6^ TC: Total cholesterol (mmol/L) ^7^ TG: Triglyceride (mmol/L). ^8^ TP: Total protein (g/L). ^9^ HDL: High-density lipoprotein cholesterol (mmol/L). ^10^ LDL: Low-density lipoprotein cholesterol (mmol/L). ^11^ VLDL: Very-low-density lipoprotein (mmol/L). ^12^ NEFA: Nonesterified Free fatty acids (umol/L). ^13^ BUN: Blood urea nitrogen (mmol/L). ^14^ Ba: Blood ammonia (umol/L). Values are presented as means ± SEM (*n* = 3). Values in the same row with the same superscript or absence of superscripts are not significantly different (*p > 0.05*). The same below.

**Table 3:** Effects of different levels of methionine on the hepatic biochemical indices of *M. Albus* after 8 weeks.

Index	M0	M2	M4	M6	M8	M10	*p* Value
^1^ HL	45.35 ± 4.22 ^a^	54.37 ± 0.79 ^b^	57.8 ± 0.92 ^bc^	62.25 ± 0.95 ^cd^	64.95 ± 1.42 ^de^	68.99 ± 0.66 ^e^	<0.001
^2^ MTTP	8.46 ± 0.41 ^c^	6.51 ± 0.11 ^b^	7.91 ± 0.15 ^c^	6.64 ± 0.25 ^b^	5.6 ± 0.41 ^a^	5.33 ± 0.1 ^a^	<0.001
^3^ Apo-A	7.64 ± 0.15 ^a^	14.31 ± 0.5 ^cd^	12.44 ± 0.73 ^b^	13.11 ± 0.25 ^bc^	13.64 ± 0.22 ^bcd^	14.89 ± 0.72 ^d^	<0.001
^4^ HSL	2.83 ± 0.06 ^d^	2.36 ± 0.02 ^c^	2.19 ± 0.08 ^c^	1.91 ± 0.03 ^b^	1.53 ± 0.03 ^a^	1.9 ± 0.1 ^b^	<0.001
^5^ LPL	1.05 ± 0.01 ^a^	1.16 ± 0.05 ^ab^	1.15 ± 0.09 ^ab^	1.22 ± 0.02 ^b^	1.39 ± 0.03 ^c^	1.16 ± 0.04 ^ab^	0.001
^6^ FAS	3.95 ± 0.03 ^a^	4.58 ± 0.06 ^b^	4.43 ± 0.06 ^b^	4.44 ± 0.34 ^b^	4.74 ± 0.09 ^b^	5.23 ± 0.08 ^c^	<0.001
^7^ ACC	9.25 ± 0.24 ^b^	7.41 ± 0.24 ^a^	7.57 ± 0.39 ^a^	7.43 ± 0.65 ^a^	7.69 ± 0.32 ^a^	6.7 ± 0.57 ^a^	0.008
^8^ CPT	1.12 ± 0.04 ^d^	1.04 ± 0.01 ^cd^	0.95 ± 0.01 ^c^	0.96 ± 0.04 ^c^	0.82 ± 0.04 ^b^	0.69 ± 0.01 ^a^	<0.001
^9^ TG	102.13 ± 1.68 ^a^	103.16 ± 3.53 ^a^	141.19 ± 3.17 ^b^	167.94 ± 1.49 ^c^	202.84 ± 3.04 ^e^	179.79 ± 3.22 ^d^	<0.001
^10^ TC	106.43 ± 1.49 ^a^	127.09 ± 3.25 ^b^	131.4 ± 1.35 ^b^	152.83 ± 2.68 ^c^	185.41 ± 1.06 ^e^	174.89 ± 3.28 ^d^	<0.001
^11^ AKP	103.05 ± 1.59 ^a^	114.37 ± 0.37 ^b^	124.59 ± 1.64 ^c^	162.96 ± 3.64 ^d^	165.64 ± 1 ^d^	163.92 ± 1.93 ^d^	<0.001
^12^ ACP	21.09 ± 0.23	21.43 ± 0.29	21.08 ± 0.18	21.8 ± 0.23	21.16 ± 0.1	21.11 ± 0.29	0.204
^13^ AST	7.22 ± 0.68 ^a^	7.89 ± 0.42 ^a^	8.47 ± 0.42 ^a^	10.19 ± 0.3 ^b^	11.54 ± 0.36 ^c^	11.78 ± 0.26 ^c^	<0.001
^14^ ALT	9.24 ± 0.45 ^a^	9.98 ± 0.35 ^a^	11.18 ± 0.29 ^b^	11.84 ± 0.36 ^b^	14.12 ± 0.21 ^c^	13.34 ± 0.61 ^c^	<0.001

^1^ HL: hepatic lipase (U/g). ^2^ MTTP: mirosomal triglygeride transfer protein (pg/mg prot). ^3^ Apo-A: Apolipoprotein -A (ug/g prot). ^4^ HSL: hormone-sensitive triglyceride lipase (U/g prot). ^5^ LPL: lipoprteinlipase (U/g prot). ^6^ FAS: fatty acid synthetase (U/g prot). ^7^ ACC: Acetyl CoA carboxylase (U/100 g prot). ^8^ CPT: carnitine palmitoyltransterase (U/g prot). ^9^ TG: Triglyceride (umol/g prot). ^10^ TC: Total cholesterol (umol/g prot). ^11^ AKP: Alkaline phosphatase (King’s unit/g prot). ^12^ ACP: Acid phosphatase (King’s unit/g prot). ^13^ AST: Aspartate aminotransferase (U/g prot). ^14^ ALT: Alanine aminotransferase (U/g prot).

**Table 4:** Effects of different levels of methionine on the contents of hepatic amino acids of *M. albus* after 8 weeks (mg/g).

Amino Acid	M0	M8	*p* Value
His ☆	1.76 ± 0.1	1.94 ± 0.06	0.179
Ser	3 ± 0.16	3.16 ± 0.07	0.422
Arg ☆	2.83 ± 0.16	2.93 ± 0.06	0.579
Gly	3.85 ± 0.18	4 ± 0.08	0.479
Asp	5.53 ± 0.29	5.75 ± 0.1	0.523
Glu	8.3 ± 0.1	8.92 ± 0.22	0.066
Thr ☆	2.95 ± 0.15	3.01 ± 0.04	0.719
Ala	4.52 ± 0.04	4.88 ± 0.13	0.064
Pro	2.92 ± 0.13	3.04 ± 0.03	0.416
Cys	0.08 ± 0	0.1 ± 0.01	0.176
Lys ☆	4.45 ± 0.21	4.65 ± 0.09	0.444
Tyr	1.26 ± 0.06	1.45 ± 0.08	0.135
Met ☆	0.88 ± 0.11	1.11 ± 0.03	0.117
Val ☆	3.57 ± 0.16	3.71 ± 0.05	0.466
Ile ☆	2.61 ± 0.12	2.62 ± 0.05	0.919
Leu ☆	4.99 ± 0.23	5.17 ± 0.08	0.496
Phe ☆	2.9 ± 0.13	2.98 ± 0.04	0.582
Total essential amino acids	26.93 ± 1.18	28.11 ± 0.42	0.399
Total non-essential amino acids	29.47 ± 0.65	31.3 ± 0.16	0.052
Total amino acids	56.39 ± 1.74	59.41 ± 0.39	0.221

* Note: ☆ essential amino acids. Values are presented as the means ± SEM (*n* = 3). Values were considered not significant at *p* > 0.05 (the same below).

**Table 5:** Effects of different levels of methionine on the contents of hepatic fatty acids of *M. albus* after 8 weeks (mg/100 g).

Fatty Acid	M0	M8	*p* Value
C14:0	1.74 ± 0.15	2.61 ± 1.11	0.516
C16:0	7.46 ± 0.4	7.2 ± 0.32	0.642
C17:0	16.11 ± 2.12	17.59 ± 6.34	0.836
C18:0	7.57 ± 0.4	7.66 ± 0.39	0.880
C23:0	19.47 ± 0.74	20.3 ± 1.2	0.587
^1^ SFAs	52.35 ± 2.6	55.36 ± 6.62	0.694
C14:1	1.95 ± 0.23	1.26 ± 0.16	0.067
C16:1	5.51 ± 0.86	8.65 ± 4.69	0.575
C18:1	22.33 ± 0.72	24.58 ± 0.68	0.086
^2^ MUFA	29.79 ± 1.41	34.49 ± 4.85	0.405
C18:2n-6	5.9 ± 0.19	6.71 ± 0.11	0.020
C20:4n-6	4.47 ± 0.66	6.49 ± 1.51	0.287
^3^ n-6 PUFA	10.37 ± 0.5	13.2 ± 1.42	0.134
C20:5n-3	3 ± 0.09	3.13 ± 0.52	0.822
C22:6n-3	28.75 ± 0.5	38.42 ± 1.17	0.020
^4^ n-3PUFA	31.75 ± 0.42	41.55 ± 1.68	0.005

^1^ SFAs: saturated fatty acids. ^2^ MUFAs: mono-unsaturated fatty acids. ^3^ n-6PUFAs: n-6 poly-unsaturated fatty acids. ^4^ n-3PUFAs: n-3 poly-unsaturated fatty acids.

**Table 6:** Composition of the diets and levels of nutrition (g/kg).

Ingredients	M0	M2	M4	M6	M8	M10
Fish meal	110	110	110	110	110	110
Soy protein concentrate	400	400	400	400	400	400
Fish oil	40	40	40	40	40	40
^1^ DL-Methionine	0	2	4	6	8	10
Lysine	3.6	3.6	3.6	3.6	3.6	3.6
Glycine	16	14	12	10	8	6
Glutamate	4	4	4	4	4	4
^2^ Food Attractant	1	1	1	1	1	1
Wheat meal	138.4	138.4	138.4	138.4	138.4	138.4
α- starch	200	200	200	200	200	200
Brewer yeast	50	50	50	50	50	50
Choline chloride	5	5	5	5	5	5
Ca(H_2_PO_4_)_2_	20	20	20	20	20	20
^3^ Vitamin and Mineral Premix	12	12	12	12	12	12
Total	1000	1000	1000	1000	1000	1000
Proximate analysis						
Dry matter (g/kg)	922.66	925.27	928.12	928.43	923.63	924.78
Crude protein (g/kg)	445.92	443.41	458.73	447.40	451.84	450.77
Crude lipid (g/kg)	67.86	67.11	68.69	67.70	67.92	68.07
Crude ash (g/kg)	102.60	101.90	100.60	102.60	101.90	100.60
Gross energy (kJ/g)	19.10	18.86	18.74	19.17	19.25	19.10

^1^ DL-Methionine (BR, 99%) was obtained from Shanghai Yuanye Biotechnology Co., Ltd. (Shanghai, China). ^2^ Attractants: 40% betaine; 20% DMPT; 20% threonine; 10% glycine; 10% inosine-5′-diphosphate trisodium salt. ^3^ Vitamin and Mineral premix was provided by MGOTer Bio-Tech Co.Ltd (Qingdao, Shandong, China)—premix composition (mg/kg diet): KCl, 200 mg; KI(1%), 60 mg; CoCl_2_·6H_2_O (1%), 50 mg; CuSO_4_·5H_2_O, 30 mg; FeSO_4_·H_2_O, 400 mg; ZnSO_4_·H_2_O, 400 mg; MnSO_4_·H_2_O, 150 mg; Na_2_SeO_3_·5H_2_O (1%), 65 mg; MgSO_4_·H_2_O, 2000 mg; Zeolite power, 3645.85 mg; VB_1_, 12 mg; Riboflavin, 12 mg; VB_6_, 8 mg; VB_12_, 0.05 mg; VK_3_, 8 mg; Inositol, 100 mg; Pantothenic acid, 40 mg; Niacin acid, 50 mg; Folic acid, 5 mg; Biotin, 0.8 mg; VA, 25 mg; VCP_1_, 5 mg; VE, 50 mg; VC, 100 mg; Ethoxyquin, 150 mg; wheat meal, 2434.15 mg.

**Table 7:** The contents of amino acids of experimental diets (g/kg).

Amino Acid	M0	M2	M4	M6	M8	M10
His ☆	9.787	9.629	9.926	9.727	9.996	9.768
Ser	18.942	18.519	19.070	18.690	18.904	18.570
Arg ☆	23.417	23.854	23.425	23.199	23.535	23.118
Gly	32.731	30.514	28.362	26.275	24.132	22.012
Asp	42.245	42.158	42.106	42.711	42.535	42.631
Glu	75.484	75.673	75.215	75.742	75.918	75.681
Thr ☆	15.514	15.230	15.556	15.925	15.412	15.881
Ala	19.718	19.301	19.759	19.447	19.697	19.424
Pro	20.227	19.697	20.153	20.330	20.575	20.228
Cys	1.084	1.029	1.088	1.091	1.084	1.094
Lys ☆	36.887	36.186	36.894	36.382	36.818	36.248
Tyr	9.802	9.759	9.852	9.040	9.397	9.634
Met ☆	1.860	3.781	5.920	7.739	9.609	11.525
Val ☆	18.640	18.211	18.637	18.379	18.590	18.323
Ile ☆	17.478	17.136	17.618	17.638	17.890	17.465
Leu ☆	29.125	29.612	29.267	29.666	29.493	29.420
Phe ☆	18.457	18.104	18.558	18.220	18.565	18.100
Trp	/	/	/	/	/	/

* Note: ☆ essential amino acids; Trp not detected.

**Table 8:** The contents of fatty acids in the experimental diets (mg/100 g).

Fatty Acids	M0	M2	M4	M6	M8	M10
C4:0	13.21	13.72	14.49	13.53	13.15	14.16
C8:0	5.07	5.08	4.91	5.05	5.04	5.00
C12:0	3.13	3.64	4.34	3.35	3.35	4.37
C13:0	11.13	10.39	9.71	11.29	10.32	10.14
C14:0	181.39	183.69	182.55	182.37	183.62	182.57
C14:1	2.19	2.62	2.81	2.88	2.70	2.83
C15:0	19.90	20.22	20.52	19.93	20.21	20.51
C16:0	609.04	608.96	606.58	609.36	608.55	606.84
C16:1	6.46	7.59	6.88	6.56	7.58	6.88
C17:0	12.58	13.74	13.65	12.80	13.42	13.52
C17:1	6.27	6.91	7.33	6.73	6.97	7.38
C18:0	120.68	121.92	121.78	121.68	121.97	121.80
18:1-T	16.16	16.09	17.89	16.10	16.02	17.86
C18:1N9C	415.27	410.17	418.66	413.30	410.15	418.53
18:2-T	2.74	3.35	2.45	2.73	3.34	2.46
C18:2N6C	17.35	16.63	18.71	18.34	16.86	18.12
C20:0	11.13	10.45	10.49	10.30	10.40	10.42
C20:1	25.44	27.37	27.27	23.43	27.34	27.22
C18:3N3	235.71	235.00	236.16	235.11	236.65	235.11
C20:2	10.35	10.88	10.31	10.36	10.85	10.34
C22:0	5.84	5.85	5.95	5.39	5.88	5.91
C22:1N9	197.83	197.62	194.40	197.33	197.65	196.49
C20:3N3	32.37	31.17	34.19	32.74	33.13	34.16
C20:4N6	25.20	25.82	25.45	25.57	25.18	25.40
C24:0	248.36	249.92	237.64	248.40	249.18	237.43
C20:5N3	101.77	100.98	101.88	101.17	101.90	101.89
C24:1	21.19	21.36	22.29	21.39	21.32	23.23
C22:6N3	575.88	571.14	571.93	575.90	571.16	570.93

**Table 9:** Primer sequence for q-PCR.

Gene	Forward (5′-3′)	Reverse (5′-3′)	* Accession no.	Size (bp)
^1^ *gcn2*	GGAACTCGTCCTGAACTG	TGGTGAAGAACTTGCCTAT	XM_020586241.1	298
^2^ *eif2a*	CCCCTTCCTTTGTTCGTC	GCTGAGGCTTTCTTGTTCC	XM_020621840.1	121
^3^ *srebf1*	GAAGACGCCAAGCCAAATGT	CCAGATGAGCAAAGCAGGGT	XM_020616413.1	152
^4^ *srebf2*	AGGTACAGGTCCTCCATCAACG	ATCGCCTTCCTCAGCACTCC	XM_020624958.1	101
^5^ *scap*	GATGGCAAACCAGAAGAACAAG	TCCGAGTCCACGCAGTAAGG	XM_020615524.1	141
^6^ *mttp*	AAGATGCTCCAGGCTTTGTT	TGTCAGGACCCTCTAAAATCAG	XM_020602163.1	172
^7^ *hdlbp*	CCACCCCAGACGACAAAGAC	GGCGAGCAACAAAATAACGA	XM_020609988.1	165
^8^ *ldlrap*	CAGGAAGACAAAAGCAAGAAGG	CGAGTGGGGTTACTATGAGGC	XM_020617284.1	194
^9^ *vldlr*	ACATCCGTCGTTTGGGTCTA	GTGGTAGTGTCCCCTCGTTT	XM_020601062.1	169
^10^ *lpl*	CGTTGACATCGGAGACCTGA	CAAAGACCACCTTGGACTGAG	XM_020613041.1	146
^11^ *pparγ*	TTCACAAGAAGTCCCGCA	AAAGAACAGGCAGGAAAACA	XM_020609689.1	203
^12^ *moat2*	TCTCCCTGCCTCTCTTTCA	TGTCCACTCCATAGTTGCCT	XM_020622089.1	213
^13^ *dgat2*	ACTTCCGCTTTCCCTTG	ATTCCCTGTCTCGTTATGTG	XM_020622054.1	104
^14^ *pparα*	GATGATGCCCTGGGATTTGA	AGCCTTGTCTGAGCACACCTG	XM_020601270.1	186
^15^ *hsl*	CCTGGGCTTTCAGTTTTCAC	AGGTTCTGGGTAATGCGTTC	XM_020597684.1	216
^16^ *fas*	CTGTCCGAGGCGGCATAAT	CCTGTTCCTTCCCCTTCTGG	XM_020608884.1	189
^17^ *fads2*	CAGCATCACGCTAAACCCA	GCGAAGATAAAATGTCAAGGC	GQ258116.1	261
^18^ *me1*	TCTTCTATCGGGTGCTAATGT	AGCCCTGATGTCTTTTTCC	XM_020621574.1	188
^19^ *me2*	AGGAGACCTTGGTGTTTATGG	TGGATTAGTGTGCCGTGC	XM_020593804.1	252
^20^ *acc*	TCTGACAGCGACCCCTTCT	GCCCCACACATTCTTATTGC	XM_020598745.1	136
^21^ *cpt1*	CCTGGAAGAAGCGTGTCATCAGAC	TGACTGGCAGGTGCTCCTGTATC	XM_020625222.1	168
^22^ *cpt2*	GCCATCTTCTGTCTCTGCC	AAGGACTTGTCATACCACCG	XM_020609923.1	107
^23^ *icdh*	GGGTATGATGAGCAGTGAGC	TATGGGATTGGTGGAGGTC	XM_020620011.1	127
^24^ *pfae*	AACTACCCACCGACCTTTG	ATGACCTTGTTATCCACTTCCT	GQ258117.1	239
^25^ *rpL17*	CGAGAACCCGACTAAATCA	GTTGTAGCGACGGAAAGG	XM_020587712.1	169

^1^ *gcn2*: general control non-derepressible. ^2^ *eif2a*: eukaryotic translation initiation factor 2. ^3^ *srebf1*: sterol regulatory element binding transcription factor 1. ^4^ *srebf2*: sterol regulatory element binding transcription factor 2. ^5^ *scap*: SREBF chaperone. ^6^ *mttp*: microsomal triglyceride transfer protein. ^7^ *hdlbp*: high density lipoprotein binding protein. ^8^ *ldlrap*: low density lipoprotein receptor adapter protein. ^9^ *vldlr*: very-low-density lipoprotein receptor. ^10^ *lpl*: lipoprotein lipase. ^11^ *pparγ*: peroxisome proliferators-activated receptor γ. ^12^ *mogat2*: monoacylglycerol O-acyltransferase 2. ^13^ *dgat2*: diacylglycerol acyltransferase 2. ^14^ *pparα*: peroxisome proliferator-activated receptor α. ^15^ *hsl*: hormone-sensitive lipase. ^16^ *fas*: fatty acid synthase. ^17^ *fads2*: fatty acid desaturase 2. ^18^ *me1*: malic enzyme 1. ^19^ *me2*: malic enzyme 2. ^20^ *acc*: acetyl-CoA carboxylase. ^21^ *cpt1*: carnitine palmitoyltransferase 1. ^22^ *cpt2*: carnitine palmitoyltransferase 2. ^23^ *icdh*: isocitrate dehydrogenase. ^24^ *pfae*: polyunsaturated fatty acid elongase. ^25^ *rpl17*: ribosomal protein L17, it is reference gene. * NCBI Reference Sequence.

## Data Availability

The datasets used and/or analysed during the current study are available from the corresponding author on reasonable request.
